# Whole-body bioluminescence imaging of T-cell response in PDAC models

**DOI:** 10.3389/fimmu.2023.1207533

**Published:** 2023-07-11

**Authors:** Roisin McMorrow, Giorgia Zambito, Alex Nigg, Karishma Lila, Thierry P. P. van den Bosch, Clemens W. G. M. Lowik, Laura Mezzanotte

**Affiliations:** ^1^Erasmus Medical Centre, Department of Radiology and Nuclear Medicine, Rotterdam, Netherlands; ^2^Erasmus Medical Centre, Department of Molecular Genetics, Rotterdam, Netherlands; ^3^Percuros BV, Leiden, Netherlands; ^4^Erasmus Medical Centre, Department of Pathology, Erasmus MC Cancer Institute, Rotterdam, Netherlands

**Keywords:** bioluminescence imaging (BLI), T-cell activation, PDAC - pancreatic ductal adenocarcinoma, immune checkpoint inhibitors (ICI), tumor microenvironment (TME), tumor-draining lymph nodes (TDLN)

## Abstract

**Introduction:**

The location of T-cells during tumor progression and treatment provides crucial information in predicting the response *in vivo*.

**Methods:**

Here, we investigated, using our bioluminescent, dual color, T-cell reporter mouse, termed TbiLuc, T-cell location and function during murine PDAC tumor growth and checkpoint blockade treatment with anti-PD-1 and anti-CTLA-4. Using this model, we could visualize T-cell location and function in the tumor and the surrounding tumor microenvironment longitudinally. We used murine PDAC clones that formed *in vivo* tumors with either high T-cell infiltration (immunologically ‘hot’) or low T-cell infiltration (immunologically ‘cold’).

**Results:**

Differences in total T-cell bioluminescence could be seen between the ‘hot’ and ‘cold’ tumors in the TbiLuc mice. During checkpoint blockade treatment we could see in the tumor-draining lymph nodes an increase in bioluminescence on day 7 after treatment.

**Conclusions:**

In the current work, we showed that the TbiLuc mice can be used to monitor T-cell location and function during tumor growth and treatment.

## 1 Introduction

Understanding the movement and activity of immune cells *in vivo* is an important pre-requisite in understanding how the host regulates its response to disease or specific treatment options. In the treatment of cancer, as well as with many other diseases, T-cells are considered to be key mediators of both pro- and anti-tumor immunity. Consequently, T-cells constitute a crucial element in the immunological response both in terms of the initiation and progression of cancer as well as in the fight against it (1). With respect to their anti-tumoral activities, many of the successful cancer immunotherapies brought to the clinic show the potential capacity to induce or enhance the infiltration of cytotoxic T-cells (2).

Whole body imaging can provide clinically relevant insights into the tumor and the surrounding areas, including the tumor-draining lymph nodes that are often survival sites where T-cells reside (1). From a preclinical setting, optical modalities can be used to provide information on cell location in a non-invasive manner (3). Bioluminescence imaging (BLI) is a preclinical optical imaging technique, widely used for biomedical research and oncological studies due to its sensitivity, ease of use and relatively low cost (4). It allows for whole-body imaging, providing a larger field of view and better sensitivity in comparison to fluorescence, since the background luminescence with bioluminescence is negligible (3, 4). Bioluminescent mouse models, including our established dual-color luciferase reporter mouse model, termed TbiLuc, have been developed to simultaneously study *in vivo* T-cell localization as well as their function (5–9). In our transgenic T-cell reporter mice, all T-cells constitutively express green-emitting click-beetle luciferase (CBG99), whereas expression of the red-emitting firefly luciferase (Ppy-RE9) is induced by Nuclear Factor of Activated T-cells (NFAT), such as during T-cell activation. Using spectral unmixing of the bioluminescent signals or using a luciferase specific substrate allows for multicolor bioluminescence imaging of T-cell location and function (5, 8).

In the last few decades, the application of immunotherapy as a form of cancer treatment has led to both partial and complete clinical responses. However, unlike other solid tumor types, immunotherapy treatments have largely proven to be ineffective in the treatment of pancreatic cancer. The most common form of pancreatic cancer is pancreatic ductal adenocarcinoma (PDAC) (10). PDAC accounts for around 90% of pancreatic cancer cases. PDAC is known for its often late-stage diagnosis and resistance to treatment. The immune landscape in PDAC has been the subject of intense research to identify and develop better therapeutic interventions (11). This PDAC-specific research has been accelerated by the insights gained by other researchers into the immune landscape of non-PDAC cancers; with work on immunotherapy responsive ‘hot’ tumors compared to resistant ‘cold’ tumors being particularly important in aiding the investigation into designing more effective therapeutic strategies for PDAC itself.

Our research aim is to establish an *in vivo* model which can examine, from a dynamic perspective, the location and state of the T-cells over time, with the aim of better understanding the insensitivity of PDAC to immunotherapy treatments, such as checkpoint inhibitors. In particular, we investigated the *in vivo* immunological response by imaging T-cell localization and function in the tumors and the tumor-draining lymph nodes in PDAC mouse models. For this purpose we employed specific KPC clones obtained from the genetically engineered mouse model, termed KPC which, is an established and clinically relevant PDAC model containing mutations occurring in both the KRAS and TP53 genes (12). These KPC clones have been selected/isolated depending on their CD8+ T-cell infiltration profile (13). More specifically, these clones have generated tumors that mimicked either a T-cell infiltrated ‘hot’ or non-infiltrated ‘cold’ tumor microenvironment (TME) in immunocompetent mice (13). Li et al., 2018 showed that both clones shared tumor antigens indicating that the ‘cold’ tumors could be susceptible to immune recognition once an effective T-cell response is generated (13).

The TbiLuc model is generated in C57BL/6 background allowing transplantation of syngeneic tumor cells like the KPC ‘hot’ and ‘cold’ clones. We improved the dual color imaging protocol by substituting the substrate CycLuc 1 with red-shifted AkaLumine for deeper tissue penetration and sensitivity (Ppy-RE9- 680nm peak) (14, 15). Like CycLuc 1, AkaLumine gives a negligible bioluminescent signal when paired with click beetle luciferase (16). The use of AkaLumine is a novel feature of the current research since it was not available when the model was originally tested (5).

In the current work, we show that the TbiLuc model can be used to investigate T-cell presence and function in the TME and the draining lymph nodes as well as the effect of immune therapy. It can help to optimize therapies that will induce T-cell infiltration and T-cell activation.

## 2 Materials and methods

### 2.1 Tumor cells and mice strain

Murine KPC clones that have been selected/isolated depending on their CD8+ T-cell infiltration profile have been used (13). These clones have been isolated from late-stage primary tumors from C57BL/6 background KPCY mice. The specific clones we used, 2838c3 and 6694c2, generated tumors that mimicked either the T-cell infiltrated ‘hot’ or non- or low infiltrated ‘cold’ tumor microenvironment in immunocompetent mice, respectively.

These KPC clones were cultured in complete Dulbecco’s Modified Eagle’s Medium (DMEM) (Gibco), supplemented with 10% fetal bovine serum (FBS) and 1% Penicillin-Streptomycin (PenStrep).

The TbiLuc mice were bred in the animal facility at Erasmus MC, Rotterdam, The Netherlands. The C57BL/6 mice used for *ex-vivo* tissue analysis were obtained from the Charles River Laboratory (The Netherlands). The animal protocols were approved by the Bioethics Committee of Erasmus MC, Rotterdam, The Netherlands (WP number SP2100031/94). The experiments were conducted in accordance with the national guidelines (National CCD license 17867) and regulations established by the Dutch Experiments on Animals Act (WoD) and by the European Directive on the Protection of Animals used for scientific purposes (2010/63/EU). TbiLuc mice (male and female) and C57BL/6 (female) were inoculated at 6-8 weeks of age, with 3x10^5^ KPC clones (2838c3 or 6694c2) and were provided access to food and water ad libitum and were hosted in the animal facility at Erasmus MC, Rotterdam, The Netherlands.

### 2.2 *In vitro* bioluminescence imaging

The spleen was isolated from the TbiLuc mice and T-cells were isolated using an easysep mouse CD8+ T-cell isolation kit (StemCell Technologies, Vancouver, Canada). Isolated cells were seeded into 96 sterile, black welled, flat bottom, plates (Greiner). They were seeded into either wells pre-coated with anti-CD3 and anti-CD28 for activating the T-cells or non-coated wells for the naive T-cell population. All T-cells were re-suspended in IMDM media with IL2 before seeding. On the day of imaging, substrates were added to the appropriate wells. 1mM of D-Luciferin Potassium Salt (Resem, BV, The Netherlands), 0.25mM AkaLumine/TokeOni (Sigma-Aldrich, St. Louis, MO, USA) and 0.1mM Cyluc1 were added. IMDM media was added to the control wells. Imaging was performed using the IVIS Spectrum imager (Perkin Elmer) 10 minutes after the addition of the substrate using FOV C, medium binning, with 30 seconds acquisition and a series of 20nm band pass filters ranging from 520nm to 740nm.

### 2.3 Cytokine ELISA assay

‘Hot’ and ‘cold’ clones 2838c3 and 6694c2, were cultured (as explained above) and the supernatant fluid from these cells was collected in order to measure the cytokine and chemokine levels using the Proteome Profiler Mouse Cytokine Array Panel A (R&D systems, Minneapolis, USA, cat. No. ARY006). Everything was performed according to the manufacturer’s instructions and the membranes were measured using the IVIS Spectrum imager (Perkin Elmer). Experiments were repeated twice showing similar cytokine results.

### 2.4 *In vivo* experiments

KPC clones 2838c3 (‘hot’) and 6694c2 (‘cold’) with 3x10^5^ were inoculated subcutaneously into the flanks of the female C57BL/6 mice (n=6). Then when the mice were sacrificed, the tumors were removed for analysis. Subsequent experiments using KPC clones 2838c3 and 6694c2 with 3x10^5^ were inoculated subcutaneously into the flanks of both randomized male and female TbiLuc mice (n=6 per group).

For the third experiment, the flank of the female TbiLuc mice (n=6 for each group) were inoculated subcutaneously with 3x10^5^ of KPC clone 2838c3. For the combination checkpoint blockade treatment, mab anti-mouse PD-1 (CD279) (Bio X cell clone 29F.1A12) was used for *in vivo* treatment at 200µg and was injected intraperitoneally (I.P.) on days 14, 17, 21, 24 and 28. Mab anti-mouse CTLA-4 (CD152) (Bio X cell clone 9H10), also at 200µg, was injected I.P. on days 14, 17 and 21. For the control group, saline solution (PBS) was injected I.P. using the same volumes as the checkpoint blockade treatments on the same days (twice on day 14, 17, 21 and once on days 24 and 28). One mouse from each group had to be removed from the study because their tumors grew and regressed before the start of the treatment on day 14.

### 2.5 Multiplex fluorescent immunohistochemistry

The tumor tissues from the C57BL/6 female mice were fixed in 4% formalin and embedded in paraffin for immunofluorescent staining using mouse U-VUE® 4 plex assay for FoxP3, CD3, CD8 and CD4 (Ultivue). The FFPE tissue slides were stained following a kit protocol and imaged on a Zeiss AxioImager M2 microscope with a Zeiss Axiocam 305 mono 5 megapixel camera. Images were made with a 20X 0.7 NA apochromat lens resulting in a 0.345 μm pixel size. VIS 2022.09 (Visiopharm®) was used for image analysis based on AI (DAPI nuclear detection, U-Net) and threshold settings (antibody fluorescence signal).

### 2.6 *In vivo* whole body bioluminescence imaging

We employed a dual color imaging protocol following intraperitoneal injection with 150mg/kg D-Luciferin (CBG99-540nm peak) and 50mg/kg AkaLumine (Ppy-RE9- 680nm peak). D-Luciferin and AkaLumine were sequentially injected in the mice (with a 4h wash out period) in order to image *in vivo* T-cell infiltration and their activation on the IVIS Spectrum imager (Perkin Elmer). For T-cell imaging, anesthetized mice were imaged 10 minutes after injection of D-Luciferin. For active T-cell imaging, mice were anaesthetized four hours after the D-Luciferin was injected and pre-scanned to ensure D-Luciferin had been cleared from the mice. Then the mice were injected I.P. with 50mg/kg AkaLumine, and imaged after 15 minutes. Imaging was performed using the IVIS Spectrum imager (Perkin Elmer) using FOV C, medium binning. The images taken at 540nm were used to quantify the D-Luciferin signal and images taken at 680nm were used to quantify the specific activation signal. To quantify the signal, specific regions of interest (ROIs) were used in a fixed size and position throughout the experiment. The background signal was corrected by subtracting the signal from the same sized ROIs placed in random positions. The residual D-Luciferin signal from the pre-scan images were subtracted from the activation signal produced after injection of Akalumine.

### 2.7 Statistical analysis

All statistical analysis was performed using GraphPad Prism 9.0 software. For mIF total counts of cells were normalized to the area considered, the means between the ‘hot’ and the ‘cold’ population of cells were compared using unpaired T test, p value <0.05 was considered significant. For *in vivo* imaging data imaging data curves were compared using Two Way Anova followed by Bonferroni-Dunn method for multiple mean comparison, p value *<0.05. **<0.01.

## 3 Results

### 3.1 AkaLumine can be used to specifically identify activated T-cells *in vitro* and is superior to CycLuc 1

**Figure 1:**
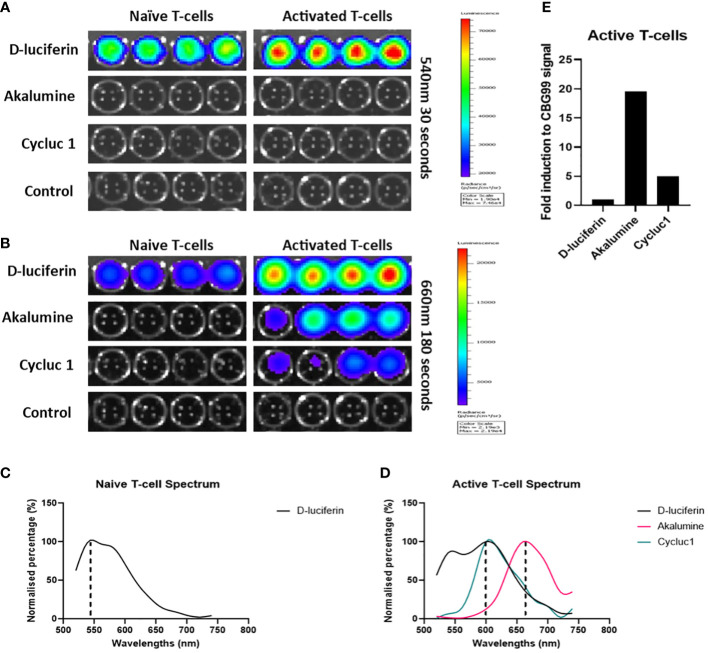
AkaLumine can be used to specifically identify activated T-cells *in vitro* and is superior to CycLuc 1. Bioluminescent expression in naïve and activated T-cells using substrates D-Luciferin, AkaLumine, CycLuc 1 and control (media alone) emission recorded 10 minutes after addition of substrates at 540nm for 30 seconds **(A)** and 660nm for 30 seconds **(B)** on the IVIS, representative data (n=1). **(C)** Normalized emission spectra of CD2- luciferase CBG99 with D-Luciferin substrate **(D)** Normalized emission spectra of CD2- luciferase CBG99 and NFAT-luciferase Ppy-RE9 with D-Luciferin, AkaLumine and CycLuc 1 substrates. **(E)** Fold induction of NFAT-luciferase expression in CD3CD28 activated TbiLuc isolated T-cells corrected to CD2- luciferase CBG99 signal in naïve T-cells.

To optimize the TbiLuc model to visualize the tumor microenvironment in black-furred mice we wanted to investigate utilizing the substrate AkaLumine. The TbiLuc mice contain a bicistronic construct that contains two luciferases CBG99 and Ppy-RE9. CBG99 is tagged to CD2 which is constitutively expressed whereas the Ppy-RE9 is driven by the hCD2 promoter (5). *In vivo* the NFAT-induced red luciferase Ppy-RE9 has a relatively weak emission. Previously the CycLuc 1 substrate was used to improve the amount of light produced but the peak emission of CycLuc 1 overlaps with D-Luciferin, the substrate used for CBG99 (5). In order to separate the signals more effectively and distinguish the differences, we wanted to employ a newer substrate AkaLumine that has been further shifted into the red spectrum (15). AkaLumine had not been developed during the time this model was developed. Like CycLuc 1, AkaLumine does not react efficiently with click beetle luciferase. To verify that this substrate can be used for our model, we tested *in vitro* isolated T-cells from the spleen of the TbiLuc mice (**Figure 1**). We induced T-cell activation in some of the naïve T-cells by using αCD3/αCD28 antibodies. We tested D-Luciferin, AkaLumine, CycLuc 1 and control with media both in naïve and activated T-cells. **Figure 1**, represents the results of emission at 540nm and 660nm respectively (**Figures 1A, B**). At 540nm, high emission signals were detected using D-Luciferin in both naïve and activated wells but were undetectable in controls, AkaLumine and CycLuc1 (**Figure 1A**). At 660nm, we detected higher emission in active T-cells with AkaLumine and CycLuc 1 but were undetectable in the naïve T-cell wells (**Figure 1B**). The spectrum of emission detected from the naïve T-cells using D-Luciferin, peaked at around 540nm whereas no peak of emission was detected using the other substrates (**Figure 1C**). The spectrum of emission of active T-cells peaked at 600nm when using the D-Luciferin and Cycluc1 substrates and at 660nm when using the AkaLumine substrate (**Figure 1D**). To calculate the fold of induction in active T-cells, we corrected the signal of Ppy-RE9/AkaLumine for the CBG99 emission signal and it resulted in a 20 fold difference for D-Luciferin whereas for Cycluc1 it resulted in a 5 fold difference (**Figure 1E**). From these results it is clear that using AkaLumine and imaging at 660nm *in vitro* (680nm *in vivo*) offer an advantage in detecting active T-cells status since it shows no detectable interference from CBG99 emission. From this we determined we could use AkaLumine to look specifically at active T-cells not only *in vitro* but most probably also *in vivo*. The advantage of using the adapted protocol is that we can optimize the visualization *in vivo* in black-furred mice.

### 3.2 ‘Hot’ KPC clone show higher cytotoxic T-cells in the tumor microenvironment than in ‘cold’ KPC clone.

**Figure 2:**
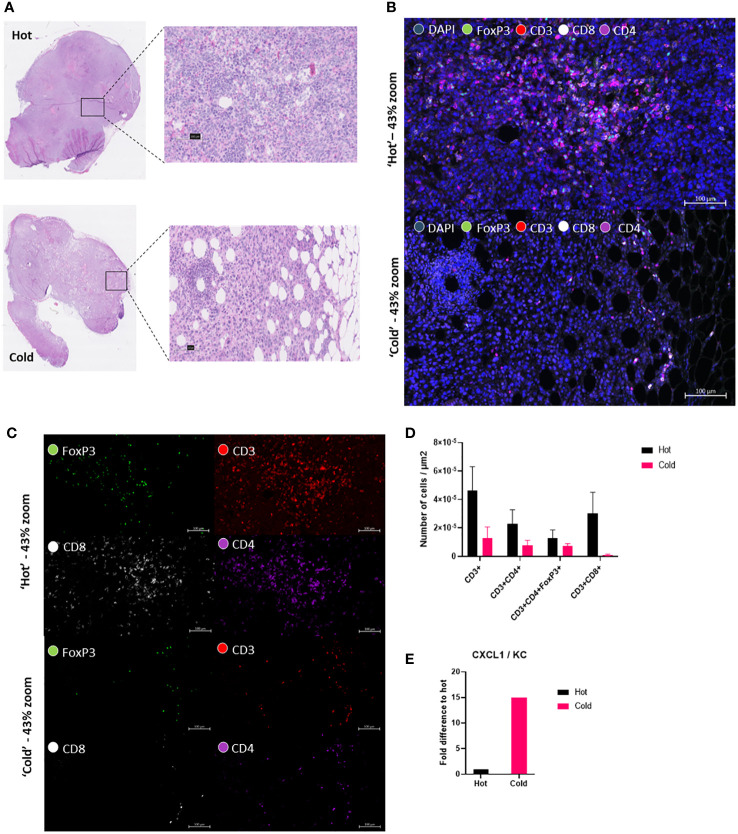
‘Hot’ KPC clone shows higher cytotoxic T-cells in the tumor microenvironment than in the ‘cold’ KPC clone. **(A)** Hematoxylin and eosin (H&E) tissue section from both ‘hot’ 2838c3 tumor slice (above) and ‘cold’ 6694c2 tumor slice (below). Figure also shows a ‘zoomed-in’ view of the region of the tumor used in the fluorescent images. **(B)** Multiplex immunofluorescent images of zoomed in area shown in H&E with DAPI nuclear stain (blue), FoxP3 (green), CD3 (red), CD8 (white), and CD4 (purple) in ‘hot’ 2838c3 (top) and ‘cold’ 6694c2 (below) tissue section shown here as a composite image of the above mentioned markers. **(C)** Same images seen in **(B)** but without DAPI stain to show the four different individual markers. **(D)** Image analysis based on AI (DAPI nuclear detection, U-Net) and threshold settings (antibody fluorescence signal) providing the counts of the different markers in selected ROI’s. These were corrected for the total ROI area and for the plotted counts from ‘hot’ 2838c3 and ‘cold’ 6694c2 tumor slices. The total counts of cells were normalized to the area considered. The means between the ‘hot’ and ‘cold’ populations of cells were compared using an unpaired T test, p<0.05 was considered significant. **(E)** Proteome profiler cytokine array immunoblot incubated with the supernatant of murine ‘hot’ 2838c3 and ‘cold’ 6694c2 KPC clones cultured in vitro. The graph displays the chemokine CXCL1/KC fold difference of Total Fluorescent Flux [p/s] of ‘cold’ 6694c2 to ‘hot’ 2838c3.

To further confirm the differences between the ‘hot’ and ‘cold’ KPC clones, which Li et al., 2018 had already investigated in great detail, we performed a multiplex cytokine ELISA looking at 40 different cytokines *in vitro*. After culturing ‘hot’ and ‘cold’ KPC clones and collecting the supernatant, we found 9 cytokines showed expression on the membrane: G-CSF, GM-CSF, sICAM-1/CD54, IP-10/CXCL10/CRF-2, CXCL1/KC, M-CSF, JE/CCL2/MCP-1, MIP-2/CXCL2, and TIMP-1 (**Supplementary Figure 1A**). This could confirm that the ‘cold’ clone had a higher fold difference of CXCL1/KC to the ‘hot’ clone (**Figure 2E**). The ‘hot’ clone also showed a higher fold difference of sICAM-1/CD54, M-CSF, JE/CCL2/MCP-1, and MIP-2/CXCL2 (**Supplementary Figure 1B**). To characterize the differences of the ‘hot’ and ‘cold’ clones’ *ex-vivo*, we performed multiplex immunofluorescence (mIF) to look at the differences in the tissue immune landscape (**Figure 2B, C**). We looked at markers CD3, CD4, CD8 and FoxP3. From this we calculated that the ‘hot’ tumors had a trend of higher numbers of CD3+ and cytotoxic T-cells, CD3+CD8+ cells (**Figure 2D**). There was no statistical difference found with both T helper cells, CD3+CD4+ and T regulatory cells, CD3+CD4+FoxP3+ (**Figure 2D**). From these additional analyses we could visualize a clear difference between the clone types (**Figure 2**).

### 3.3 Total T-cells signal in tumor area is higher in ‘hot’ versus ‘cold’ KPC tumors, in a volume dependent way.

**Figure 3:**
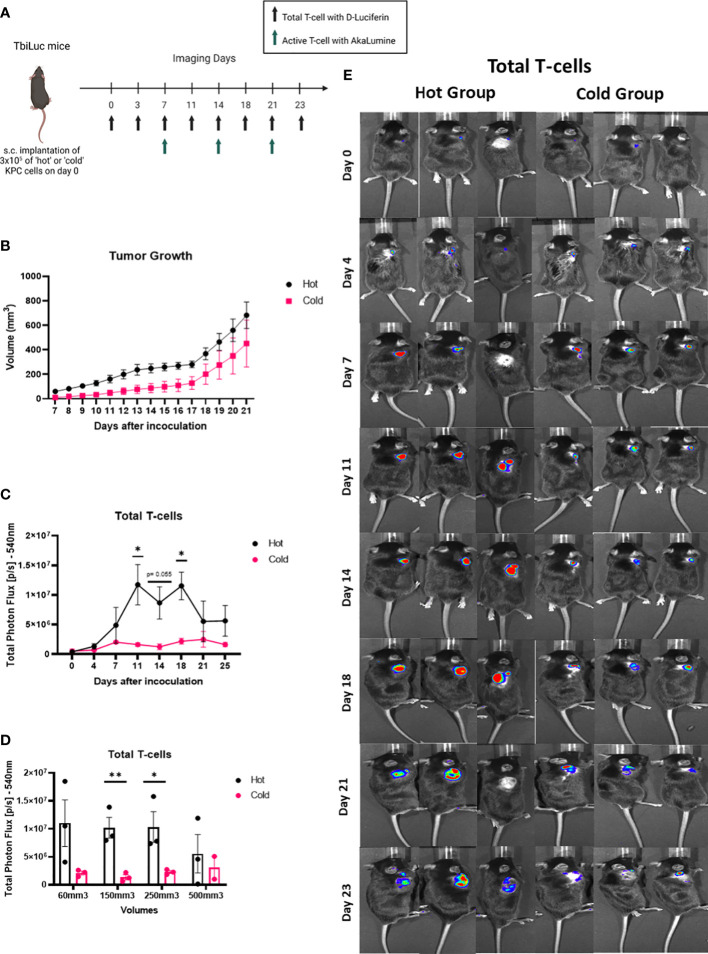
Total T-cells signal in the tumor area is higher in the ‘hot’ versus the ‘cold’ KPC tumors, in a volume dependent way. **(A)** Representative image of TbiLuc mice that were injected subcutaneously with either 3x105 of ‘hot’ 2838c3 (n=3) or ‘cold’ 6694c2 (n=3) cells on the right flank, image was created with BioRender.com. These mice were imaged, several times for 23 days after inoculation, with substrate D-Luciferin to look at the total T-cells or AkaLumine to look at the active T-cells. **(B)** Tumor volumes (mm3) were measured twice a week with a caliper and here the average volume is plotted of ‘hot’ 2838c3 (n=3) and ‘cold’ 6694c2 (n=3) tumors, represented with SEM. **(C** Total Photon Flux [p/s] at 540nm with an exposure time of 30 seconds where D-Luciferin (150mg/kg) was injected, 10 minutes prior to imaging, intraperitoneally. The mean of the total photon flux was plotted and SEM was plotted. **(D)** The total photon flux mean is plotted for when the mice had tumor volumes measured around 60, 150, 250 and 500mm3. **(E)** Images of TbiLuc mice with merged bioluminescent signal at 540nm. The mice were imaged on the dorsal side on day 0 to day 23. All images are displayed at the same intensity scale. The region of interest (ROI) was quantified around the tumor area. All the statistics produced here were calculated using Two Way ANOVA (analysis of variance) followed by the Bonferroni-Dunn method for multiple mean comparison. * p < 0.05; and ** p < 0.01. .

We then wanted to investigate if the differences between the ‘hot’ and ‘cold’ KPC tumor microenvironments could be seen *in vivo*. We inoculated subcutaneously syngeneic murine ‘hot’ KPC cells or ‘cold’ KPC cells into the flanks of both randomly assigned male and female TbiLuc mice. We imaged the mice twice a week to look at the total amount of T-cells with D-Luciferin (150 mg /kg) and the active T-cells with AkaLumine (50 mg /kg) from day 0 to day 23 (**Figures 3A, E**). We observed differences in the growth of the tumors in the mice over time where the ‘hot’ tumors grew faster than the ‘cold’ tumors (**Figure 3B**). On days 11 and 18 we saw a significantly higher signal of total photon flux in the ‘hot’ tumor region than in the ‘cold’ tumors and at day 14 the p value was 0.055 (**Figure 3C**). After day 18 the difference was lost (**Figure 3C**). When the tumors were around 150-250mm3 there was a significant difference. At around 500mm3 the difference in signal is lost between the ‘hot’ and ‘cold’ tumors (**Figures 3D, E**).When looking at the active T-cell emissions, we saw no significant difference between the ‘hot’ and ‘cold’ tumor areas (**Supplementary Figures 2A, B**).

### 3.4 Total T-cell infiltration increases in lymph nodes with the addition of checkpoint blockers anti-PD-1 and anti-CTLA-4.

**Figure 4:**
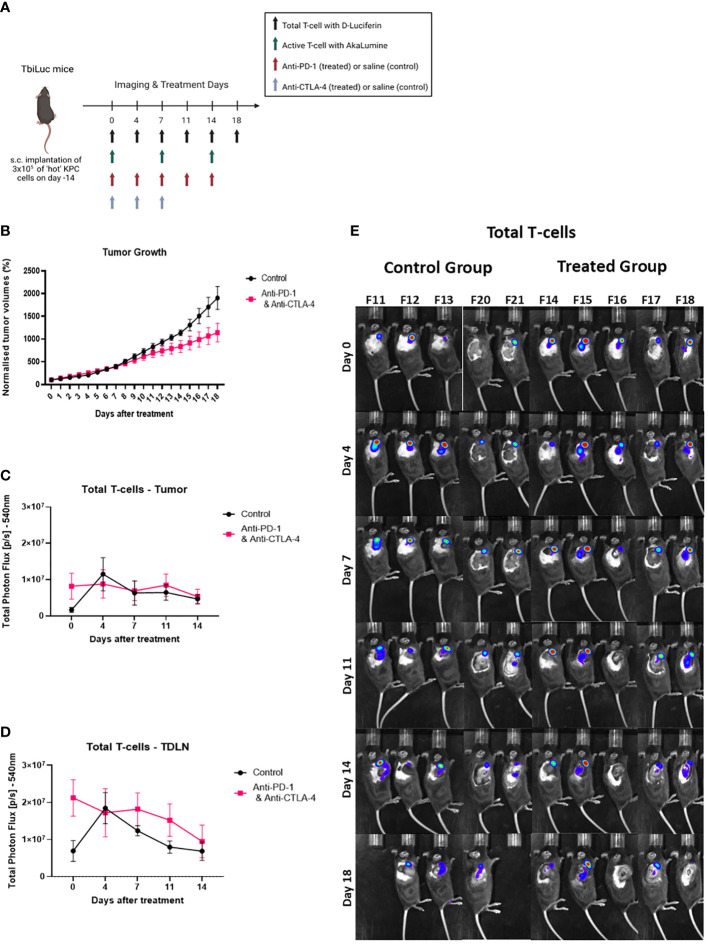
Total T-cell infiltration increases in lymph nodes with the addition of the checkpoint blockers anti-PD-1 and anti-CTLA-4. **(A)** Representative image of TbiLuc mice that were injected subcutaneously with 3x105 of ‘hot’ 2838c3 (n=3) cells on the right flank, treatment began on day 14 after injection of tumor cells,, image was created with BioRender.com. These mice were imaged several times from day 0 of treatment until day 18 of treatment with substrate D-Luciferin to look at total T-cells or with AkaLumine to look at active T-cells. **(B)** Represents normalized tumor volumes (mm3) from day 0 of treatment that were measured twice a week with a caliper of anti-PD-1 and anti-CTLA-4 treated TbiLuc mice (n=5); and control, saline treated (n=5), mice, represented with SEM. **(C)** Total Photon Flux [p/s] at 540nm with an exposure time of 30 seconds where D-Luciferin (150mg/kg) was injected, 10 minutes prior to imaging, intraperitoneally. The mean of the total photon flux of the tumor was plotted and the SEM was plotted. **(D)** Total Photon Flux [p/s] at 540nm with an exposure time of 30 seconds where D-Luciferin (150mg/kg) was injected, 10 minutes prior to imaging, intraperitoneally. The mean of the total photon flux was plotted of the tumor-draining lymph node (TDLN) and SEM was plotted **(E)** Images of TbiLuc mice with merged bioluminescent signal at 540nm on day 0 before mice were treated with anti-PD-1 and anti-CTLA-4 or PBS until day 18 of treatment, imaged on the right dorsal side to look at the tumor and the tumor-­draining lymph nodes (TDLN).

After confirming that we could use this model for investigating the differences between the ‘hot’ and ‘cold’ tumors, we then wanted to investigate if we could see differences when a treatment is introduced. Li et al., 2018 tested different therapeutic combinations and saw a significant increase in survival with anti-PD-1 and anti-CTLA-4 in mice with ‘hot’ tumors (13). We inoculated subcutaneously syngeneic ‘hot’ KPC cells into the flanks of female TbiLuc mice. On day 0, (14 days after inoculation), we randomly assigned the mice to receive either vehicle (saline) or anti-PD-1 (200µg) five times over 14 days, every 2 to 3 days, together with anti-CTLA-4 (200µg) three times over 7 days (**Figure 4A**). We saw 16 days after treatment a significant difference in the growth of tumors in the treated mice (n=5) versus the control mice (n=5) (**Figure 4B**). We investigated the total T-cells in the tumor and the tumor-draining lymph node. From this we saw no difference in the treated vs the non-treated mice in the tumor (**Figure 4C**). However, when looking at the tumor-draining lymph node (TDLN), we saw over time that there was a higher signal in the treated group p<0.03 and F=5.156 (**Figure 4D**), suggesting an active immune response.

### 3.5 Active T-cell infiltration increases in the tumor-draining lymph node 7 days after checkpoint blocker treatment

**Figure 5:**
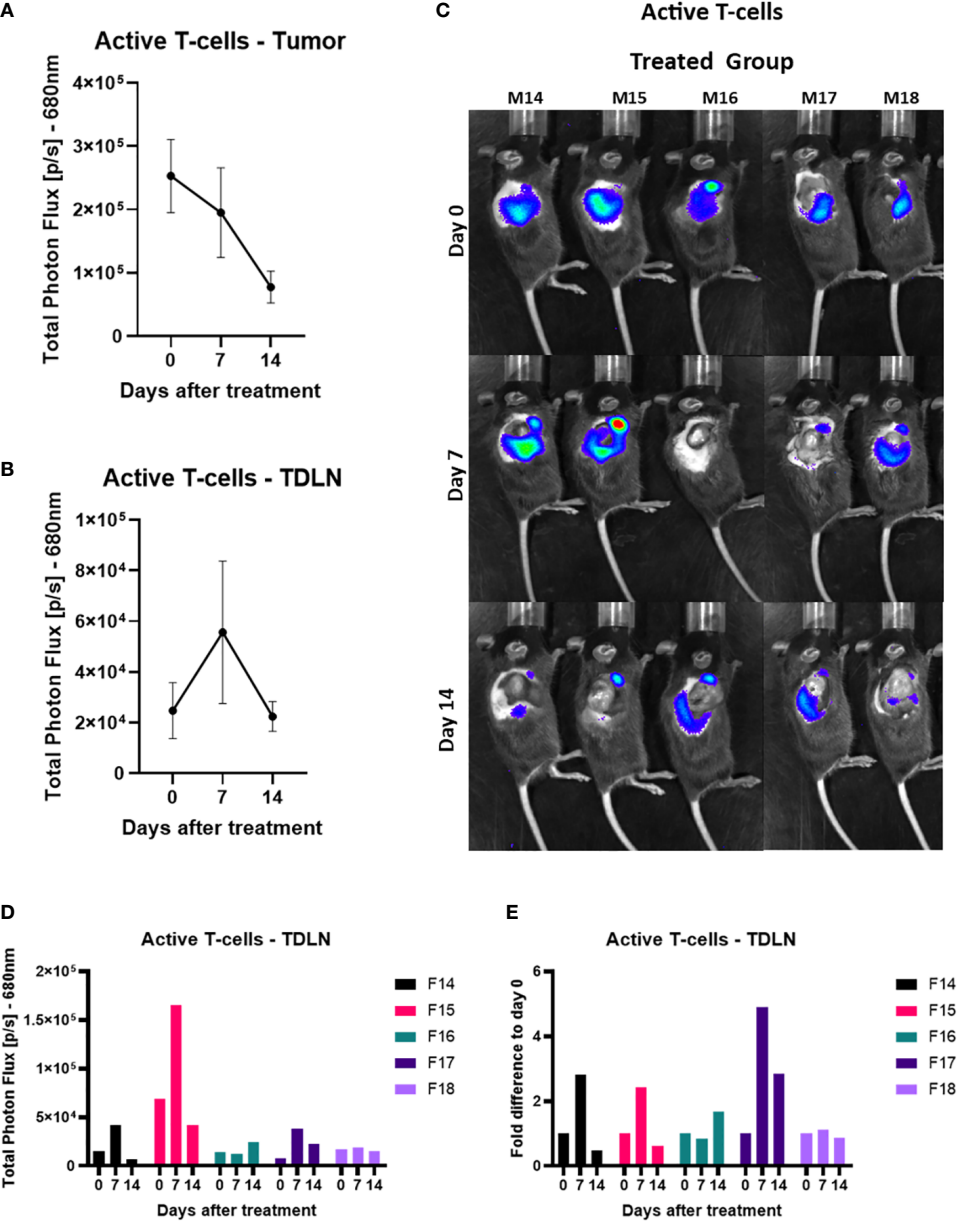
Active T-cell infiltration increases in the tumor-draining lymph node 7 days after the checkpoint blocker treatment. **(A)** Total Photon Flux [p/s] at 680nm with an exposure time of 180 seconds where AkaLumine (50mg/kg) was injected, 15 minutes prior to imaging, intraperitoneally. The mean of the total photon flux was plotted of the tumor and SEM was plotted. **(B)** Total Photon Flux [p/s] at 680nm with an exposure time of 180 seconds where AkaLumine (50mg/kg) was injected, 15 minutes prior to imaging, intraperitoneally. The mean of the total photon flux was plotted of the tumor-­draining lymph node (TDLN) and SEM was plotted **(C)** Images of TbiLuc mice with merged bioluminescent signal at 680nm on day 0 before mice were treated with anti-PD-1 and anti-CTLA-4 or PBS until day 14 of treatment, imaged on the right dorsal side to look at the tumor and the tumor-draining lymph nodes (TDLN). **(D)** Total Photon Flux [p/s] at 680nm of individually treated mice number F14 to F18 on days 0, 7, and 14 after start of treatment of anti-PD-1 and anti-CTLA-4. **(E)** Fold difference of Total Photon Flux [p/s] at 680nm from day 0 of individually treated mice number F14 to F18 on days 0, 7, and 14 after start of treatment of anti-PD-1 and anti-CTLA-4.

After looking at the total amount of T-cells, we then investigated if we could see any activity of T-cells in the anti-PD-1 and anti-CTLA-4 treated tumor area and in the TDLN which could be an early biomarker of response. There was a continuous regression of the signal over time with the emission of photons in the active T-cells in the tumor (**Figure 5A**). In the TDLNs of treated mice we saw on day seven a large variation in the signal in comparison to the other days (**Figure 5B**).We further investigated this by looking at the active T-cells in the TDLN’s in the individual mice. From this we saw that two mice (numbers 14 and 15) had a significantly increased signal in comparison to the other treated mice throughout the treatment (**Figures 5C, D**). On day 7, 3 out of the 5 mice had an increased signal compared to day 0 (numbers 14, 15 and 17); 1 out of 5 had this on day 14 (number 16); and one mouse showed no obvious difference in signal from day 0 (number 18). The signal increase in TDLN’s after therapy correlated with better tumor regression compared to controls, this is further supported by the fact that the mouse that showed no difference in signal over the two weeks (number 18) had the fastest growing tumor out of the five treated mice (**Figures 5C–E**, **Supplementary Figure 3D**)

## 4 Discussion

In this study, we demonstrated that our dual-color bioluminescence reporter mouse, TbiLuc, can be used to image, non-invasively, T-cell localization and T-cell function in PDAC. We investigated this during both the tumor growth phase and the treatment intervention phase.

We further improved the original TbiLuc imaging protocol using AkaLumine, instead of CycLuc 1, to look at active T-cells. AkaLumine is shifted further to the red spectrum. This is preferred due to lower absorption of light at longer wavelengths, with the result that we could achieve a better separated spectrum with D-Luciferin to look at the naïve/total T-cell population. The problem with CycLuc 1 is that its peak of emission overlaps with D-Luciferin’s shifted spectrum in activated T-cells. Due to a more red shifted light emission of AkaLumine we could not only achieve a better separation of spectrums but also get a deeper tissue penetration. The latter allowed us to use the TbiLuc mice for directly imaging T-cell activation instead of having to isolate the T-cells and transfer them into recipient mice (5).

Li et al., 2018 characterized, in great detail, the differences between the ‘hot’ and ‘cold’ tumors derived from the KPC clones (13). The TME of the ‘hot’ murine PDAC models are characterized by having higher infiltration levels of T-cells; whereas the ‘cold’ non or low T-cell infiltrated clones have higher infiltration levels of immune suppressive MDSC’s which in turn block the infiltration of T-cells. Of course, the presence of T-cells alone in the tumor cannot always achieve tumor regression but they do aid the achievement of durable remissions, as demonstrated in the ‘hot’ clones when T-cells were depleted (13).

We could confirm *in vitro* that the ‘cold’ clone secreted higher levels of CXCL1 than ‘hot’ clone which was described as the major distinguishable element between the ‘hot’ and ‘cold’ murine PDAC tumors. The presence of tumor derived CXCL1 was linked to the upregulating of immunosuppressive granulocytic MDSCs into the tumor (13). *Ex-vivo* we could confirm using multiplex immunofluorescence that the ‘hot’ tumors had more cytotoxic T-cells than the ‘cold’ tumors (**Figure 2**).

*In vivo* we saw a higher amount of total T-cells from day 11 using BLI, confirming the feasibility of this imaging technique to appreciate differences reflecting *ex–vivo* analysis. This signaling difference persists for 10 days and is lost from day 21 onwards. When we investigated the signal at similar tumor volumes, we discovered that from around a volume of 150-250mm3 we see a significant difference in the signal which is then lost when the tumors reach a size of around 500mm3. This loss in the signaling difference is most likely due to decreased light penetration in thicker tumors but could also be due to hypoxia, necrosis or pH changes in the tumor that can influence the bioluminescent read out (17). These results indicates that the application of the bioluminescence imaging modality should be limited to a certain volume range.

In the treatment experiment with anti-PD-1 together with anti-CTLA-4 versus control (saline), we observed a slightly higher signal at day 7 in the tumor of the treated versus the control but from day 11 the signals in the treated versus control were similar. In general, the efficacy of the treatment on tumor growth was limited in this model and it is reflected in the fact that we could not detect an increased infiltration of T-cells into the tumor which is one of the proposed criteria for predicting rates of survival in patients receiving ICIs (18). In general, many tumor cell lines that are responsive to checkpoint therapy are characterized by dynamic changes occurring during tumor development. This is particularly evident with respect to the immune infiltration populations of activated cytotoxic T-cells linked to an effective cytolytic T-cell immune response seen in the CT 26 cell line (19).

One of the big advantages of TbiLuc is its ability to perform whole body imaging in the mice. Using BLI can provide high sensitivity to small populations on the cellular and subcellular levels at a low cost and it can be imaged longitudinally. It can be combined with a modality like PET/SPECT that can provide additional information regarding where the T-cells are in the tumor; as well as being more translatable to clinical settings (20–22). Currently the available methods to monitor responses to immunotherapy in the clinic are mainly based on *ex vivo* analyses of tissues or of blood (23). Bensch et al., 2018 found that when comparing patient response predictions with their *in vivo* PET tracer versus IHC or RNA seq. they found their imaging modality to be more predictive (24). One widely used method for assessing baseline tumor metabolism and glycolytic activity is 18F-FDG PET/CT. Using FDG PET has been a crucial tool in evaluating responses in clinical studies to immunotherapy. FDG-PET has been shown to be useful in identifying adverse events in organs that are immunotherapy related, as well as predicting patient response (25–28). For example, in a preclinical study, Le et al., 2023, used FDG PET to visualize systemic effects, stimulated by STING agonist-induced lymphocytes in KPC tumor bearing mice and found an increased uptake of 18F-FDG in secondary lymph nodes (29).

By imaging on the right dorsal side, in the treatment experiment, we could use TbiLuc to look not only at the tumor but also at the axial lymph node next to the tumor which can be considered as a tumor-draining lymph node (TDLN) (**Figures 4**, **5**) (30). Imaging T-cells in secondary lymphoid organs, using targeted imaging techniques such as those employed by TbiLuc, can significantly improve a model’s ability to infer, and possibly predict, the success rates of specific treatments (23, 29, 31, 32). For example, using CD8+ T-cell radiolabeled with 89Zr, Alsaid and colleagues found that the number of CD8 T-cells increased in the TDLN’s, already 4 days after the start of ICOS/anti-PD-1 treatment, and in the tumor 11 days after (23). This CD8 minibody is now in phase 2 clinical trials in patients treated with immune checkpoint blockers (NCT03802123 and NCT05013099) (33, 34). The recent literature also suggests that tumor specific T-cells coming from the TDLNs are crucial to antitumor immunity and it appears that TDLN’s are enriched with these types of T-cells (35, 36). Nagasaki et al., 2022 found that the addition of anti-PD-1 therapy promotes the infiltration, into the TME, of tumor-attacking exhausted T-cells clonotypes from the TDLN’s. In other cases, increased T-cell presence in the TDLN’s have been detected several times in the literature to occur when tumor cells are treated with checkpoint blockers (23, 29, 31, 36, 37). In particular, imaging active T-cells, using an anti-OX40 radiotracer, in a model of cancer treated with CpG vaccination/adjuvant therapy, clearly showed the TDLN persistent enhanced signal throughout the treatment period (on days 2 and 9 after treatment) (38). Chen et al., 2022 (9) also developed a way to image Granzyme B activity with bioluminescence (22).

In our study, when looking at the TDLN signals, TbiLuc imaging showed the presence and increased signal of active T-cells at day 7 after treatment. The observed variation in signals is reflective of the biological variability concerning the differences in infiltration of immune cells also seen in the human population (18). Importantly, we observed that mice having a high and sustained signal during the treatment, especially at day 7 after commencement of treatment, were subsequently correctly predicted to respond best to the treatment. Further investigations will be aimed at characterizing T-cells in tumors and TDLNs via *ex vivo* analysis of tumors and TDLNs, which was not the primary goal of this study and it would have required sacrificing mice at different time points. LaSalle et al. showed that the moderately immunogenic CT26 colon cancer KRAS mutant showed activation signals in the tumor-draining lymph node as a biomarker of early response after ICI combination therapy of the same antibodies (anti-PD-1 and anti-CTLA-4) (39). In addition, the study suggested that the TDLN has a prominent role in the response to low immunogenic tumors compared to highly immunogenic ones (40).

## 5 Conclusions

The future goal of our ongoing research is to improve the efficacy of immunotherapy for PDAC. One way of doing this would be through looking at the specific treatments which can improve the infiltration and/or activation of T-cells in the tumors and the TDLNs. In this context, the use of AkaLumine as a substrate in the TbiLuc mice improved the use of the model. AkaLumine may aid in the identification of immunotherapy interventions that can increase infiltration of T-cells in the TDLN’s and most importantly into the tumor in PDAC, where tumor volume remains a critical factor to be considered.

## Data availability statement

The original contributions presented in the study are included in the article/**Supplementary Material**. Further inquiries can be directed to the corresponding author.

## Ethics statement

The animal protocols were approved by the Bioethics Committee of Erasmus MC, Rotterdam, The Netherlands (WP number SP2100031/94). The experiments were conducted in accordance with the national guidelines (National CCD license 17867) and regulations established by the Dutch Experiments on Animals Act (WoD) and by the European Directive on the Protection of Animals used for scientific purposes (2010/63/EU).

## Author contributions

RM, LM, GZ, CL, contributed to conception and design of the study. RM, KL, performed experiments. RM, AN, LM analyzed the data. RM, LM performed the statistical analysis. RM wrote the first draft of the manuscript. All authors contributed to the article and approved the submitted version.

## Supplementary material

Supplementary Figure 1 **(A)** Images of the proteome profiler cytokine array immunoblot incubated with the supernatant of murine ‘hot’ 2838c3 (immunoblot K0333) and ‘cold’ 6694c2 (immunoblot K0335) KPC clones, cultured in vitro IVIS. **(B)** The graph displays the chemokines sICAM-1/ CD54; M-CSF; JE/ CCL2/ MCP-1; and MIP-2/ CXCL2 fold difference of Total Fluorescent Flux [p/s] of ‘hot’ 2838c3 to ‘cold’ 6694c2.

Supplementary Figure 2 **(A)** Images of TBiLuc mice with merged bioluminescent signal at 680nm after I.P. injection of Akalumine (50mg/kg), at day 7, 14 and 21; and after injection subcutaneously of either ‘hot’ or ‘cold’ KPC clones. **(B)** Total Photon Flux [p/s] at 680nm with an exposure time of 180 seconds. The mean of the Total photon flux was plotted of the tumor area and the SEM was plotted.

Supplementary Figure 3 **(A)** Total Photon Flux [p/s] at 680nm with an exposure time of 180 seconds. The mean of the Total photon flux was plotted of the tumor and the SEM was plotted. **(B)** Total Photon Flux [p/s] at 680nm with an exposure time of 180 seconds. The mean of the Total photon flux was plotted of the tumor draining lymph node and the SEM was plotted. **(C)** Images of TBiLuc mice with merged bioluminescent signal at 680nm after I.P. injection of Akalumine (50mg/kg), at day 0, 7 and 14; and after the start of the treatment (saline solution – control group). **(D)** Represents normalized tumor volumes (mm3) from day -7 after start of the treatment. It was measured twice a week with a caliper of individual measurements of control saline treated mice (n=5, above) and anti-PD-1 and anti-CTLA-4 treated TbiLuc mice (n=5, below) and, represented with the SEM.
