# Supplementary figures and images for: Whole-body bioluminescence imaging of T-cell response in PDAC models
Source: Front Immunol. 2023 Jul 11;14:1207533. doi: 10.3389/fimmu.2023.1207533 (PMC10367003; doi:10.3389/fimmu.2023.1207533)

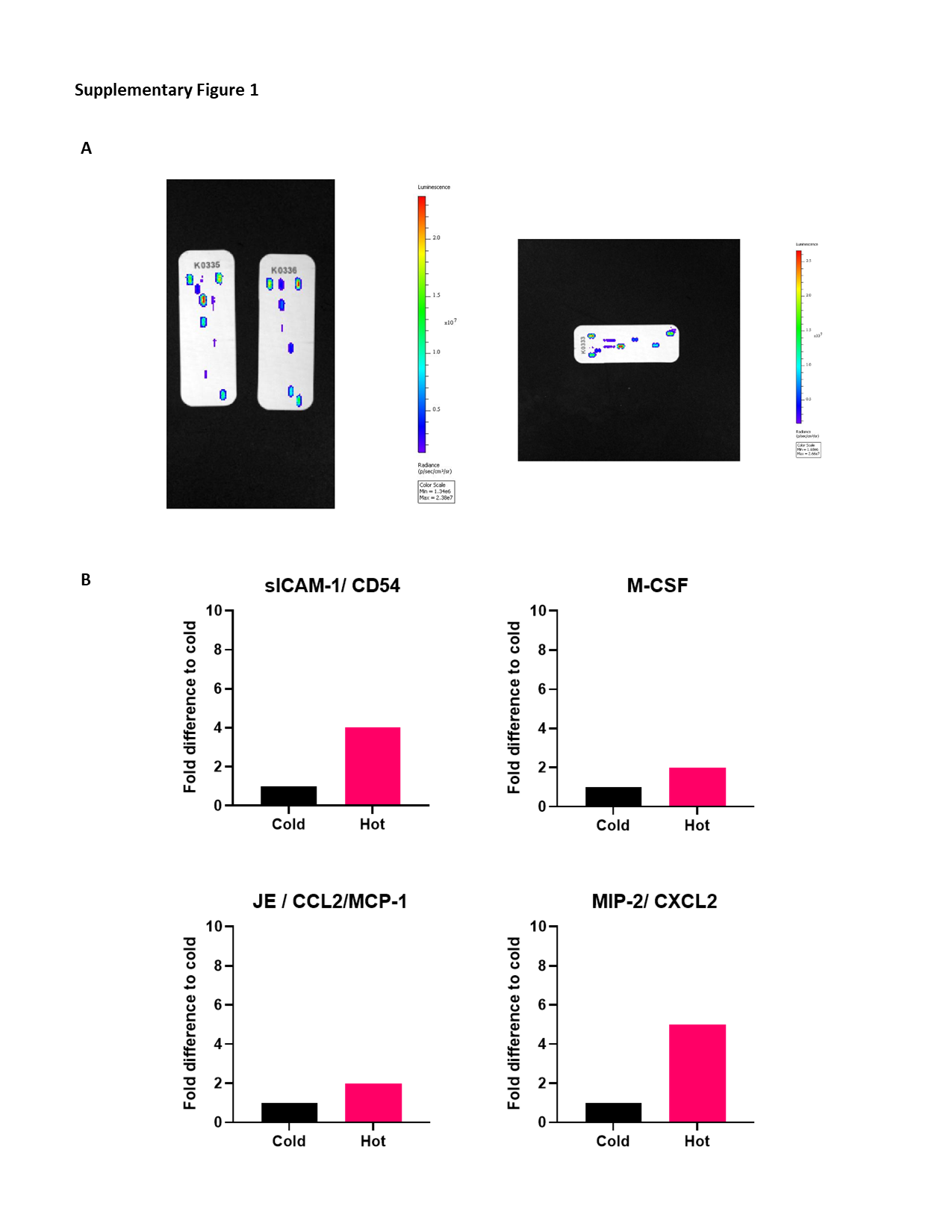

Supplement: Supplementary Figure 1 — (A) Images of the proteome profiler cytokine array immunoblot incubated with the supernatant of murine ‘hot’ 2838c3 (immunoblot K0333) and ‘cold’ 6694c2 (immunoblot K0335) KPC clones, cultured in vitro IVIS. (B) The graph displays the chemokines sICAM-1/ CD54; M-CSF; JE/ CCL2/ MCP-1; and MIP-2/ CXCL2 fold difference of Total Fluorescent Flux [p/s] of ‘hot’ 2838c3 to ‘cold’ 6694c2. [file Image_1.tif]

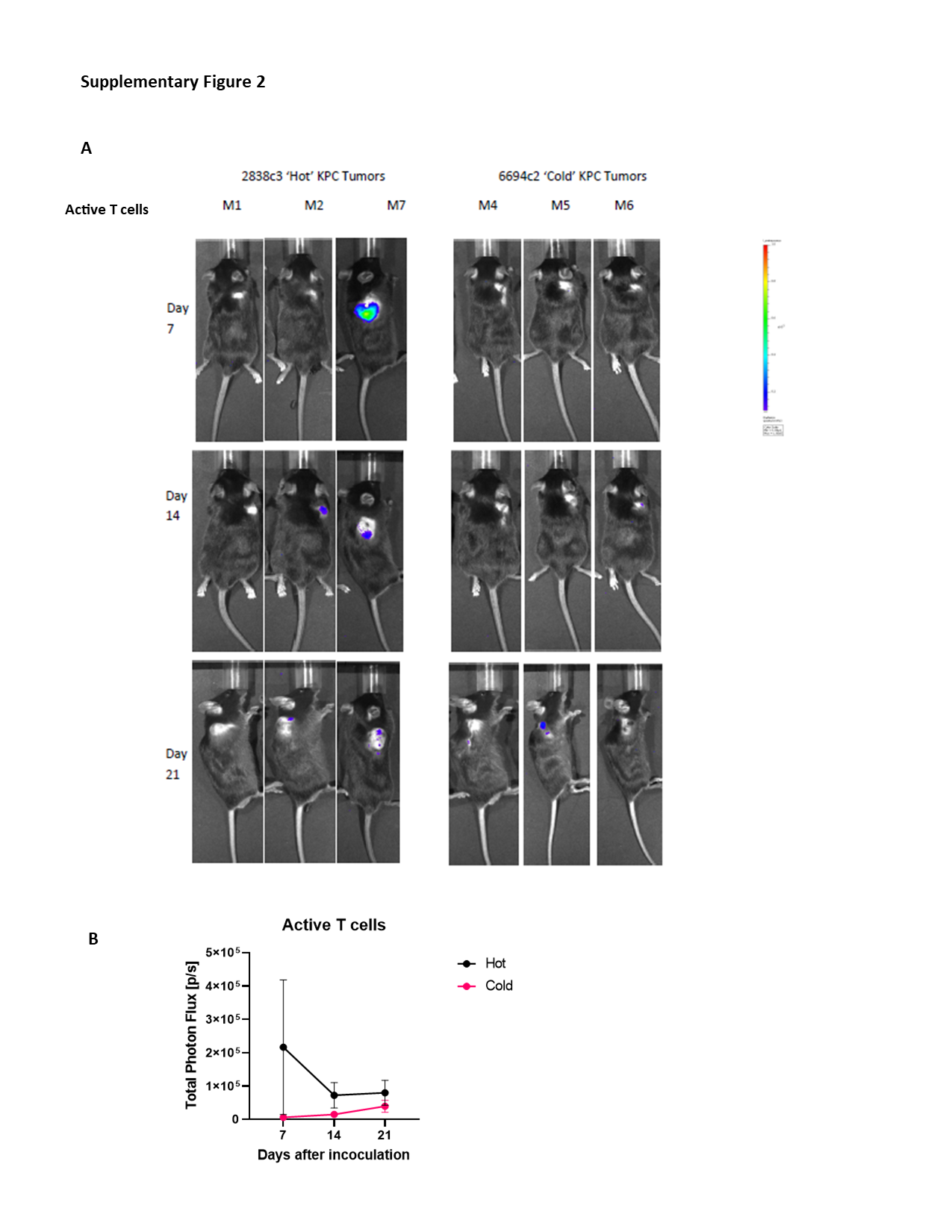

Supplement: Supplementary Figure 2 — (A) Images of TBiLuc mice with merged bioluminescent signal at 680nm after I.P. injection of Akalumine (50mg/kg), at day 7, 14 and 21; and after injection subcutaneously of either ‘hot’ or ‘cold’ KPC clones. (B) Total Photon Flux [p/s] at 680nm with an exposure time of 180 seconds. The mean of the Total photon flux was plotted of the tumor area and the SEM was plotted. [file Image_2.tif]

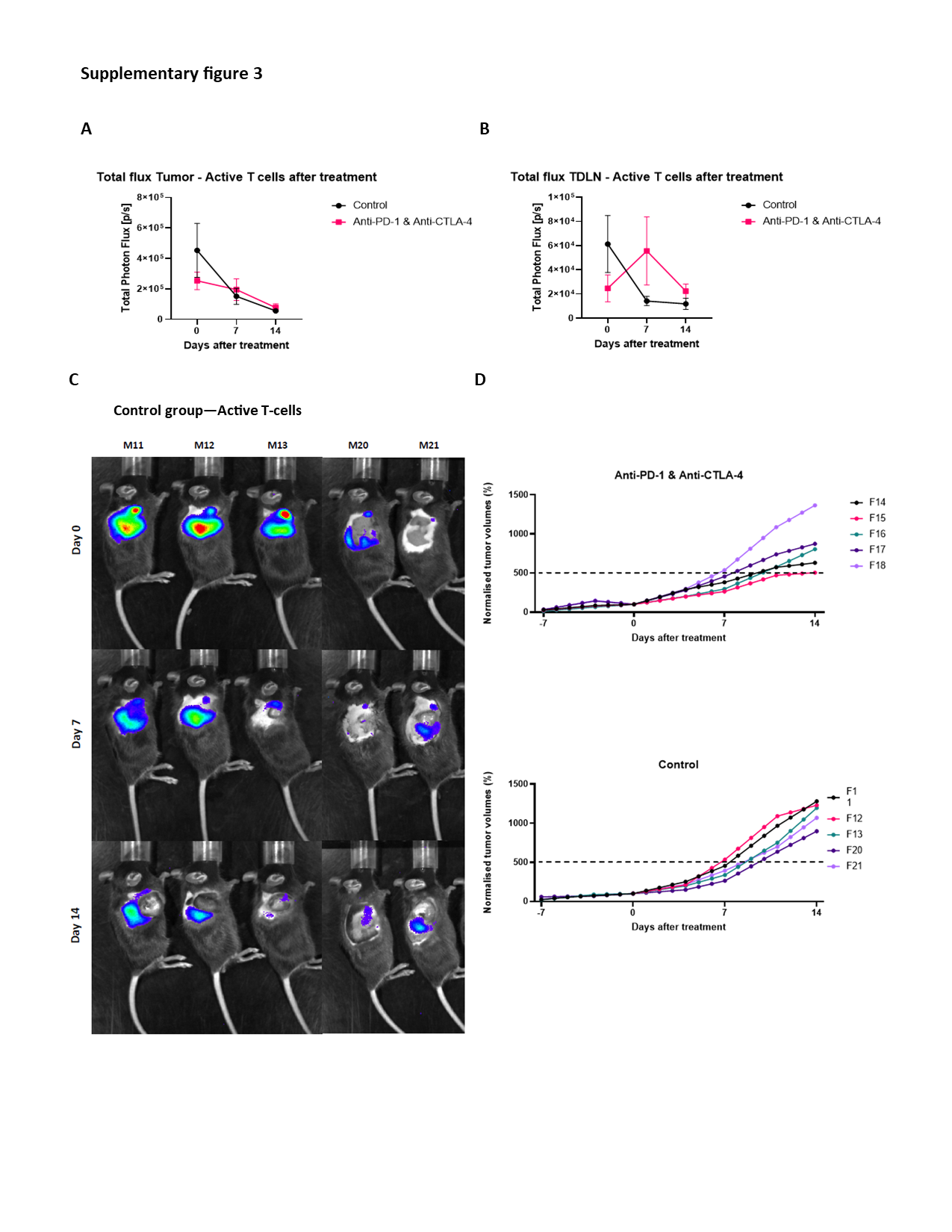

Supplement: Supplementary Figure 3 — (A) Total Photon Flux [p/s] at 680nm with an exposure time of 180 seconds. The mean of the Total photon flux was plotted of the tumor and the SEM was plotted. (B) Total Photon Flux [p/s] at 680nm with an exposure time of 180 seconds. The mean of the Total photon flux was plotted of the tumor draining lymph node and the SEM was plotted. (C) Images of TBiLuc mice with merged bioluminescent signal at 680nm after I.P. injection of Akalumine (50mg/kg), at day 0, 7 and 14; and after the start of the treatment (saline solution – control group). (D) Represents normalized tumor volumes (mm3) from day -7 after start of the treatment. It was measured twice a week with a caliper of individual measurements of control saline treated mice (n=5, above) and anti-PD-1 and anti-CTLA-4 treated TbiLuc mice (n=5, below) and, represented with the SEM. [file Image_3.tif]
